# Attenuation of a Virulent Porcine Deltacoronavirus Strain DHeB1 via Serial Passage in LLC-PK1 Cells

**DOI:** 10.3390/v17050695

**Published:** 2025-05-12

**Authors:** Yuhan Zhang, Kang Liu, Longfei Chen, Meng Yuan, Hongyu Lu, Shaobo Xiao, Liurong Fang

**Affiliations:** 1 National Key Laboratory of Agricultural Microbiology, College of Veterinary Medicine, Huazhong Agricultural University, Wuhan 430070, China; zyhsh47@webmail.hzau.edu.cn (Y.Z.); ddrothschild@163.com (K.L.); clf96@webmail.hzau.edu.cn (L.C.); yuanmeng@webmail.hzau.edu.cn (M.Y.); lu-hy@webmail.hzau.edu.cn (H.L.); vet@mail.hzau.edu.cn (S.X.); 2 Key Laboratory of Preventive Veterinary Medicine in Hubei Province, Cooperative Innovation Center for Sustainable Pig Production, Wuhan 430070, China

**Keywords:** Porcine deltacoronavirus (PDCoV), pathogenicity, immunogenicity, genetic variation analysis

## Abstract

Porcine deltacoronavirus (PDCoV) is a newly discovered enteropathogenic coronavirus primarily responsible for diarrhea and mortality in piglets, with the potential to infect humans, thereby posing a significant threat to both human health and the global pig industry. Currently, there is no commercially available live-attenuated vaccine for PDCoV. In this study, an isolated virulent PDCoV strain, DHeB1, was continuously passaged in LLC-PK1 cells for up to 110 passages. The virus growth kinetics in cell culture and complete genome sequences of various passages (F11, F40, F70, F90, and F110) were determined. The results indicated significant increases in virus titers at passages F40 and F90. Sequence analysis revealed that only a few single-nucleotide mutations (some of which resulted in amino acid changes) and one nucleotide insertion were observed throughout successive passages. Notably, the eight and seven amino acid mutations that emerged in F40 and F70, respectively, remained stable in subsequent passages and were predominantly located in the S glycoprotein. The pathogenicity of F11, F40, F70, and F90 was assessed in 5-day-old piglets, revealing markedly reduced clinical symptoms, histopathological lesions, and intestinal PDCoV antigen distributions in piglets inoculated with F70 or F90. Importantly, F90 exhibited little to no virulence in piglets. The immunogenicity of F70, F90, and F110 was further evaluated in weaned piglets, with results indicating that the neutralizing antibody titers induced by F70 and F90 were comparable and significantly higher than those induced by F110. Collectively, these findings suggest that the PDCoV strain DHeB1 has been attenuated and can be used to develop a live-attenuated vaccine against PDCoV.

## 1. Introduction

Porcine deltacoronavirus (PDCoV) is a novel enterovirus belonging to the genus Deltacoronavirus within the family *Coronaviridae*. Under the electron microscope, PDCoV particles exhibit spherical or elliptical morphology, with diameters ranging from 80 to 160 nm, featuring corona spikes on their envelope [1]. The PDCoV genome is an unsegmented single-stranded positive-sense RNA approximately 25.4 kb in length. It comprises 5′ and 3′ untranslated regions (UTRs) and contains at least nine open reading frames (ORFs) that encode 15 mature nonstructural proteins (nsp2 to nsp16), 4 structural proteins (spike protein S, envelope protein E, membrane protein M, and nucleocapsid protein N), and 3 accessory proteins (NS6, NS7, and NS7a) [2,3].

Pigs of all ages are susceptible to PDCoV, especially piglets within 7 days of age. Infected piglets typically exhibit symptoms such as anorexia, watery diarrhea, vomiting, and dehydration. The infection rate of PDCoV can reach 100%, with a mortality rate of 40% to 80% among infected piglets. This virus has emerged as a significant pathogen associated with mortality in piglets [4,5,6]. Clinically, PDCoV frequently co-infects with other porcine diarrhea viruses, including porcine epidemic diarrhea virus (PEDV) and transmissible gastroenteritis virus (TGEV). Such co-infections lead to more severe clinical disease and complicate the diagnosis, prevention, and control of these infections [7]. Current research confirms that PDCoV can spread across species, infecting not only pigs but also other animals such as chickens, turkeys, and mice [8]. Furthermore, Lednicky et al. detected the PDCoV genome in plasma samples from children with fever in Haiti and successfully isolated the virus, suggesting that PDCoV may be a potential zoonotic pathogen capable of infecting humans [9].

PDCoV was initially identified in 2012 during molecular surveillance of mammalian and avian coronaviruses in Hong Kong [10]. The outbreak of PDCoV in swine herds was first reported in the United States in 2014. To date, the occurrence and prevalence of PDCoV have been documented in at least 11 countries or regions worldwide, including China, Canada, Korea, Japan, and Thailand [11,12,13,14,15]. Based on the complete genome sequences, PDCoV strains can be categorized into four distinct lineages: Chinese, Early Chinese, the USA, and Southeast Asian [16]. The strains within the USA lineage were primarily isolated from the USA, Japan, and Korea, along with some strains from China (CHN-GD16-05) [17]. The Chinese lineage predominantly includes the currently circulating strains in mainland China and exhibits a 3-nucleotide (AAT) deletion in the S gene compared with the USA lineage [18,19]. The Early Chinese lineage is represented by strains such as CHN-AH-2004, HKU15-44, and HKU15-155 [20]. The Southeast Asian lineage comprises strains circulating in countries such as Laos, Vietnam, and Thailand, as well as two strains recently discovered in Guangxi, China (CHN/GX/1468B/2017 and CHN-GX81-2018) [21,22]. Since the outbreak of PDCoV, it has not only hindered the sustainable growth of the global pig industry but has also posed a significant threat to human life and health. Vaccination plays a crucial role in the prevention and control of PDCoV. Currently, although several types of PDCoV vaccines are under investigation, including inactivated virus vaccines [23], subunit vaccines [24], and viral vectored vaccines [25], few commercial vaccines have been released. Inactivated virus vaccines and subunit vaccines are the most widely utilized vaccine types; however, both require multiple immunizations and adjuvants to enhance the immune responses. Meanwhile, viral vectored vaccines have emerged as focal points of scientific interest. Nevertheless, the capacity of viral vectors to carry genes is limited, making it difficult to accommodate large foreign gene fragments. In contrast, live-attenuated vaccines are promising due to their strong immunogenicity and lower immunization costs. A common method for obtaining attenuated virus strains involves serially passaging field viruses in vitro. Many attenuated virus strains, such as PEDV [26], porcine reproductive and respiratory syndrome virus (PRRSV) [27], and severe acute respiratory syndrome coronavirus 2 (SARS-CoV-2) [28], have been successfully obtained using this traditional method.

In this study, the PDCoV strain DHeB1, isolated by our laboratory, was passaged in LLC-PK1 cells for up to 110 passages. We investigated the virus growth kinetics in cell culture and the genetic variation among different passaged strains (F11, F40, F70, F90, and F110). The pathogenicity of strains F11, F40, F70, and F90 was evaluated in 5-day-old piglets via clinical assessment, histopathology, and immunohistochemical analysis. Furthermore, the immunogenicity of strains F70, F90, and F110 was evaluated in weaned piglets. The findings of this study will lay the foundation for the development of safe and effective PDCoV vaccines.

## 2. Materials and Methods

### 2.1. Ethics Statement

All animal experiments conducted in this study were approved by the Committee on the Ethics of Animal Experiments of Huazhong Agricultural University (Ethics Approval Number: HZAUSW-2021-0014; HZAUSW-2022-0032).

### 2.2. Cell Culture and Virus

LLC-PK1 cells were purchased from the American Type Culture Collection (ATCC CL-101; Manassas, VA, USA) and cultured in Minimum Essential Medium (MEM, HyClone, Logan, UT, USA) supplemented with 10% fetal bovine serum (FBS, Transgen Biotech, Beijing, China) at 37 °C in a humidified atmosphere with 5% CO_2_. The passage of PDCoV was performed using LLC-PK1 cells as previously described [29]. Briefly, the culture medium was removed from 90% confluent LLC-PK1 cell monolayers in 6-well culture plates, and the cells were washed twice with sterile phosphate-buffered saline (PBS, 0.01 M, pH 7.2). Subsequently, the prepared virus was added to the monolayer cells and incubated at 37 °C in 5% CO_2_ for 2 h. After incubation, the inoculum was removed, and 2 mL of MEM containing 7.5 µg/mL trypsin (Gibco, Grand Island, NE, USA) was added. When more than 80% of the cells exhibited cytopathic effects (CPEs), the supernatants and cells were harvested and stored at −80 °C until use.

### 2.3. Immunofluorescence Assay (IFA)

LLC-PK1 cells were cultured in 12-well plates and infected with DHeB1-F11, F40, F70, F90, or F110 at a multiplicity of infection (MOI) of 0.01. At 15 h post-infection (hpi), the cells were fixed with 4% paraformaldehyde for 15 min and subsequently permeabilized with methanol for another 15 min at room temperature. After three washes with PBST (PBS containing 0.05% Tween-20), the cells were blocked with PBS containing 5% BSA for 1 h at 37 °C. A mouse anti-PDCoV-N protein monoclonal antibody, prepared in our laboratory, was then added and incubated at 37 °C for 1 h. Following this, the cells were washed three times with PBST and incubated with an Alexa Fluor^®^ 488-conjugated goat anti-mouse IgG (H+L) antibody (Abcam, Cambridge, UK) for 1 h at 37 °C. Finally, the cell nuclei were counterstained with 4′, 6-diamidino-2-phenylindole (DAPI, Beyotime Biotechnology, Shanghai, China), and the stained cells were examined using an inverted fluorescence microscope (Olympus, Tokyo, Japan) under dark conditions.

### 2.4. Growth Kinetics

The growth kinetics of PDCoV DHeB1-F11, F40, F70, F90, and F110 were established as previously described with some modifications [30]. Briefly, LLC-PK1 cells were inoculated with DHeB1-F11, F40, F70, F90, or F110 at 0.01 MOI. The cell cultures were harvested at 6, 12, 18, 24, 30, 36, 42, and 48 hpi, respectively. After two rounds of freeze-thawing, cell debris was removed by centrifugation, and the viral titer of each sample was determined using the 50% tissue culture infectious dose (TCID_50_) assay in LLC-PK1 cells.

### 2.5. Complete Genome Sequencing and Phylogenetic Analysis

The complete genomes of PDCoV DHeB1-F11, F40, F70, F90, and F110 were amplified using twenty pairs of specific primers, as previously described. The amplified fragments were then subjected to Sanger sequencing. The genomic fragments were assembled using SeqMan software in DNAstar Lasergene 7.0. Sequence alignment analysis was performed using the Clustal W program within MEGA 11.0 software. Additionally, phylogenetic analysis of the PDCoV DHeB1 passaged strains (F11, F40, F70, F90, and F110) and 49 reference PDCoV strains (Table S1) was conducted using MegAlign in DNAstar Lasergene 7.0 software. A phylogenetic tree based on the complete genome sequences of these strains was constructed using the neighbor-joining method with 1000 bootstrap replications in MEGA 11.0 software.

### 2.6. Pathogenicity of PDCoV DHeB1 Serially Passaged Strains in 5-Day-Old Piglets

To investigate the pathogenicity of various passages of PDCoV DHeB1, twenty-five 3-day-old piglets were obtained from a farm with no previous history of PEDV infection, and the sows were confirmed as negative for PDCoV, PEDV, and TGEV antigens and antibodies through RT-PCR and serum neutralization test (SNT) prior to delivery. The piglets were randomly assigned to five groups (*n* = 5) and housed in separate rooms. At 5 days old, the piglets in four groups were each orally inoculated with 2 mL (2 × 10^8^ TCID_50_) of PDCoV DHeB1 serially passaged strains (F11, F40, F70, F90), respectively, while the remaining five piglets received the same volume of MEM as a mock treatment. Clinical symptoms were observed daily, and fecal consistency was scored using the following criteria: 0 = solid, 1 = pasty, 2 = semi-liquid, and 3 = liquid. Rectal swabs were collected from all piglets at 1, 3, 5, and 7 days post-challenge (dpc), and the copies of PDCoV RNA were quantified using SYBR Green real-time RT-qPCR as previously described. One piglet from each group was randomly selected for euthanasia and necropsy examination at 60 h post-challenge (hpc).

### 2.7. Histopathology and Immunohistochemistry

At necropsy, small intestinal segments, including the duodenum, jejunum, and ileum, were collected from each piglet. These segments were fixed in 4% paraformaldehyde for 48 h and subsequently embedded in paraffin. The paraffin-embedded tissues were then sectioned using a microtome (Leica, Wetzlar, Germany) and placed on slides. Following deparaffinization and hydration, the tissues were stained with hematoxylin and eosin (H&E) for histopathological examination or subjected to immunohistochemistry (IHC) using a PDCoV N-specific monoclonal antibody prepared in our laboratory.

### 2.8. Immunogenicity of PDCoV DHeB1 Serially Passaged Strains in Weaned Piglets

To assess the immunogenicity of PDCoV DHeB1 serially passaged strains, twenty weaned piglets, all negative for PEDV, PDCoV, and TGEV antigens and antibodies, were obtained from a swine farm. These piglets were randomly divided into four groups (*n* = 5), including a mock group and three immunization groups. At 30 days old, the piglets in the immunization groups were intramuscularly vaccinated with 2 mL (2 × 10^6^ TCID_50_) of PDCoV DHeB1 serially passaged strains (F70, F90, F110), while the mock group received 2 mL of MEM. Serum samples were collected weekly, and the neutralizing antibodies against PDCoV were determined using a serum neutralization test.

### 2.9. Serum Neutralization Test

Sera were inactivated at 56 °C for 30 min and then serially two-fold diluted starting at a dilution of 1:4. Subsequently, the diluted serum was mixed with an equal volume of 200 TCID_50_ of the PDCoV DHeB1 strain and incubated for 1.5 h at 37 °C. The culture medium was discarded from the 90% confluent LLC-PK1 cell monolayers in 96-well culture plates, and the cells were washed twice with sterile PBS. The serum–virus mixture was then inoculated onto the LLC-PK1 cell monolayers (100 μL/well). After 1.5 h of incubation at 37 °C with 5% CO_2_, the inoculum was removed, and the cells were washed twice with sterile PBS. Following this, 100 μL of MEM containing 7.5 µg/mL of trypsin was added to each well. The plates were cultured at 37 °C with 5% CO_2_ for 3–4 days. The titer of the neutralizing antibody against PDCoV was calculated according to the Reed–Muench method.

### 2.10. Statistical Analysis

Statistical analyses were performed using GraphPad Prism 8.0 software, employing either one-way or two-way ANOVA as appropriate. Data are presented as mean ± standard error of the mean (SEM). A *p*-value of less than 0.05 was deemed statistically significant (* *p* < 0.05; ** *p* < 0.01; *** *p* < 0.001).

## 3. Results

### 3.1. Biological Characteristics of PDCoV DHeB1 Serially Passaged Strains

**Figure 1:**
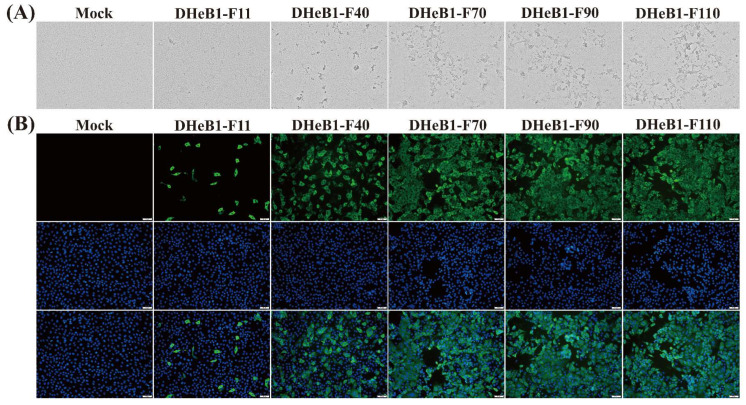
Biological characteristics of PDCoV DHeB1 serially passaged strains. (**A**) Cytopathic effects of LLC-PK1 cells infected with 0.01 MOI of PDCoV DHeB1 serially passaged strains (F11, F40, F70, F90, or F110) at 15 hpi. (**B**) Immunofluorescence analysis of LLC-PK1 cells infected with PDCoV DHeB1 serially passaged strains (F11, F40, F70, F90, or F110). The PDCoV DHeB1 serially passaged strains (F11, F40, F70, F90, or F110) were inoculated into LLC-PK cells at an MOI of 0.01. At 15 hpi, LLC-PK cells were stained with a mouse anti-PDCoV N protein monoclonal antibody and an Alexa Fluor^®^ 488-conjugated goat anti-mouse IgG (H+L) secondary antibody (green fluorescence). Nuclei were counterstained with DAPI (blue fluorescence).

**Figure 2:**
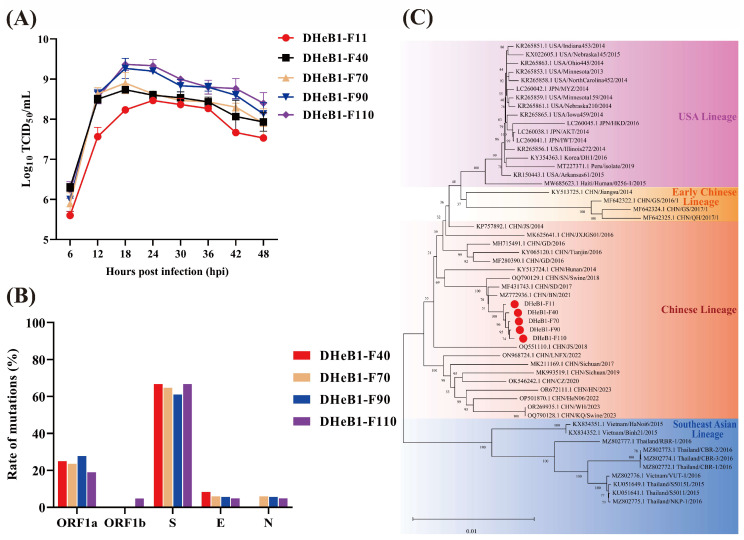
Growth kinetics, phylogenetic, and genetic variation analysis of PDCoV DHeB1 serially passaged strains. (**A**) Growth kinetics of PDCoV DHeB1 serially passaged strains (F11, F40, F70, F90, and F110) in LLC-PK1 cells. LLC-PK1 cells were inoculated with selected passages of PDCoV DHeB1 at 0.01 MOI, and the cell cultures were harvested at designated time points (6, 12, 18, 24, 30, 36, 42, and 48 hpi). The viral titers were determined using the 50% tissue culture infectious dose (TCID_50_) assay. (**B**) Mutation rates of various proteins encoded by PDCoV DHeB1 serially passaged strains. The amino acid sequences of PDCoV DHeB1 serially passaged strains were aligned with PDCoV DHeB1-F11. The mutation rate of each viral protein (ORF1a, ORF1b, S, E, and N) was determined by calculating the proportion of mutated amino acids relative to the total number of amino acids within that protein. (**C**) A phylogenetic analysis of five PDCoV DHeB1 passaged strains (F11, F40, F70, F90, and F110) alongside 49 reference PDCoV strains (Table S1) was conducted based on complete genome sequences. The phylogenetic tree was constructed using the neighbor-joining method implemented in MEGA 11.0 software. Bootstrap analysis was performed with 1000 replicates, and the resulting bootstrap values are displayed on each branch. The scale bar represents nucleotide substitutions per site. The red dots indicate PDCoV DHeB1 serially passaged strains.

The biological characteristics of the PDCoV DHeB1 strain during serial passage in vitro were investigated by inoculating LLC-PK1 cells with selected passages (F11, F40, F70, F90, and F110) at 0.01 MOI. A CPE, characterized by shrunken, rounded, and aggregated cells, was observed at 15 hpi. The cells inoculated with high-passage strains (F70, F90, and F110) exhibited more pronounced shrinkage and detachment from the cell monolayer compared to those inoculated with low-passage strains (F11 and F40) (Figure 1A). The five passaged strains were further confirmed by IFA using a mouse anti-PDCoV-N protein monoclonal antibody. At 15 hpi, green fluorescent signals were detected in cells infected with PDCoV DHeB1 F11, F40, F70, F90, or F110, while no green fluorescence was observed in uninfected cells. Furthermore, nearly all cells infected with high-passage strains (F70, F90, and F110) displayed green fluorescence, whereas only a limited number of cells infected with the low-passage strain (F11) showed green fluorescence (Figure 1B). Growth kinetics analysis revealed that the viral titer of PDCoV DHeB1 increased gradually during serial passage in vitro, accompanied by a reduction in the time required to reach peak titer. The viral titers of F40, F70, F90, and F110 peaked at 18 hpi, while F11 peaked at 24 hpi, 6 h later than the high-passage strains (Figure 2A). These findings suggest a gradual adaptation of PDCoV DHeB1 to LLC-PK1 cells with successive passage.

### 3.2. Complete Genome Sequencing and Phylogenetic Analysis of PDCoV DHeB1 Serially Passaged Strains

To analyze the genetic variation in PDCoV DHeB1 during serial passage in vitro, we determined and analyzed the complete genome sequences of F11, F40, F70, F90, and F110. The results indicated that F40, F70, F90, and F110 exhibited 12, 17, 18, and 21 amino acid (aa) alterations, respectively, compared to F11. These aa mutations were distributed across ORF1a, ORF1b, S, E, and N proteins, with the majority (61.7–66.7% of the total altered aa) concentrated in the S glycoprotein. (Figure 2B and Table 1). No mutations were detected in the M protein. Additionally, a single nucleotide insertion (409C410) was identified in the 5′UTR of F70, F90, and F110; however, no nucleotide insertions or deletions were noted in F40. Notably, the eight and seven aa mutations observed in F40 and F70, respectively, which were primarily concentrated in the S glycoprotein, remained stable throughout subsequent passage. Further sequence homology analysis revealed that PDCoV DHeB1 shared a nucleotide identity of 97.8% to 99.8% with strains identified in China, the USA, Japan, and Korea, while displaying a slightly lower identity of 97.1% to 97.7% with strains from Thailand and Vietnam (Figure S1). Phylogenetic analysis, based on the complete genome sequences of DHeB1 serially passaged strains and 49 reference PDCoV strains, indicated that all PDCoV strains form four distinct lineages: Chinese lineage, Early Chinese lineage, USA lineage, and Southeast Asian lineage. The DHeB1 strain clustered within the Chinese lineage, suggesting that DHeB1 may share a common evolutionary ancestor with strains from this lineage (Figure 2C).

### 3.3. Clinical Evaluation of Piglets Challenged with PDCoV DHeB1 Serially Passaged Strains

**Figure 3:**
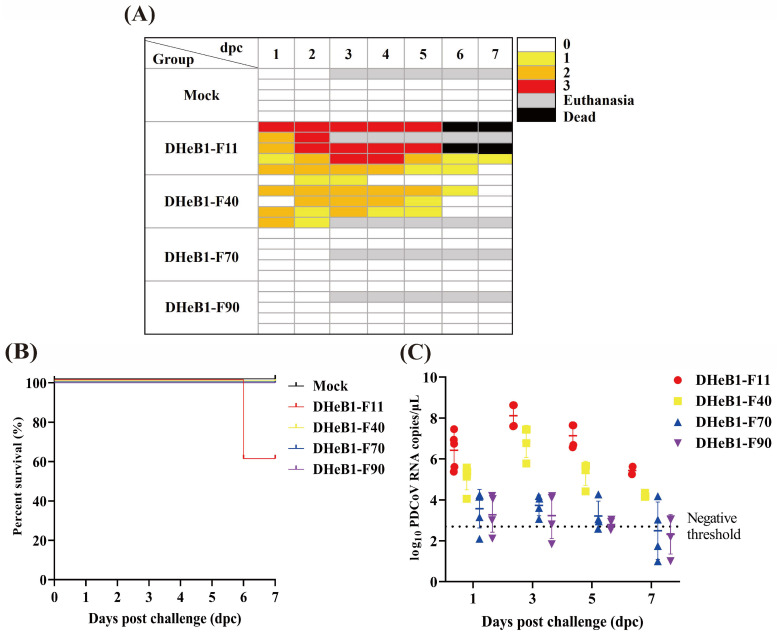
Clinical observations and fecal virus shedding detection of piglets inoculated with PDCoV DHeB1 F11, F40, F70, or F90. (**A**) Fecal scores of piglets post-challenge were categorized as FS = 0, 1, 2, and 3, which correspond to solid feces, pasty feces, diarrhea with semi-liquid feces, and watery diarrhea with liquid feces, respectively. (**B**) Kaplan–Meier curves for survival rates of piglets orally challenged with PDCoV DHeB1 F11, F40, F70, or F90. (**C**) Fecal virus shedding in 5-day-old piglets challenged with PDCoV DHeB1 F11, F40, F70, or F90. Negative threshold: less than 10^2.7^ copies/μL is considered negative.

To evaluate potential alterations in the virulence of the PDCoV DHeB1 strain during serial passaging in vitro, the F11, F40, F70, and F90 passages were selected for oral inoculation of 5-day-old piglets. As anticipated, all piglets in the PDCoV DHeB1-F11 challenge group developed clinical signs by 1 dpc. The most severe clinical symptoms were observed between 2 and 5 dpc, characterized by watery or semi-liquid feces (score 2–3), accompanied by vomiting, anorexia, and lethargy. By 6 dpc, two of the five piglets had succumbed to PDCoV DHeB1-F11 infection, while the remaining piglets gradually recovered (Figure 3A,B). Moderate clinical symptoms were noted in piglets inoculated with PDCoV DHeB1-F40. Three of the five piglets developed diarrhea by 1 dpc, and all five exhibited mild to moderate diarrhea (score 1–2) between 2 and 5 dpc. Notably, none of the piglets in this challenge group experienced vomiting or mortality. Throughout the experiment, piglets in the mock, F70, and F90 challenge groups remained active, with no typical clinical signs observed (Figure 3A). PDCoV RNA shedding in fecal swab samples was detected using RT-qPCR. The piglets inoculated with PDCoV DHeB1-F11 exhibited the highest viral shedding titers, reaching a peak at 3 dpc. In the DHeB1-F40 infected group, the viral loads in fecal swab samples similarly peaked at 3 dpc, but the titers were significantly lower than those in the DHeB1-F11 group. Notably, viral shedding was detected in only some of the piglets inoculated with DHeB1-F70 or F90, and the titers were significantly lower than those observed in piglets inoculated with DHeB1-F11 or F40 (Figure 3C). No PDCoV RNA was detected in samples from piglets in the mock group.

### 3.4. Histopathological Observations

**Figure 4:**
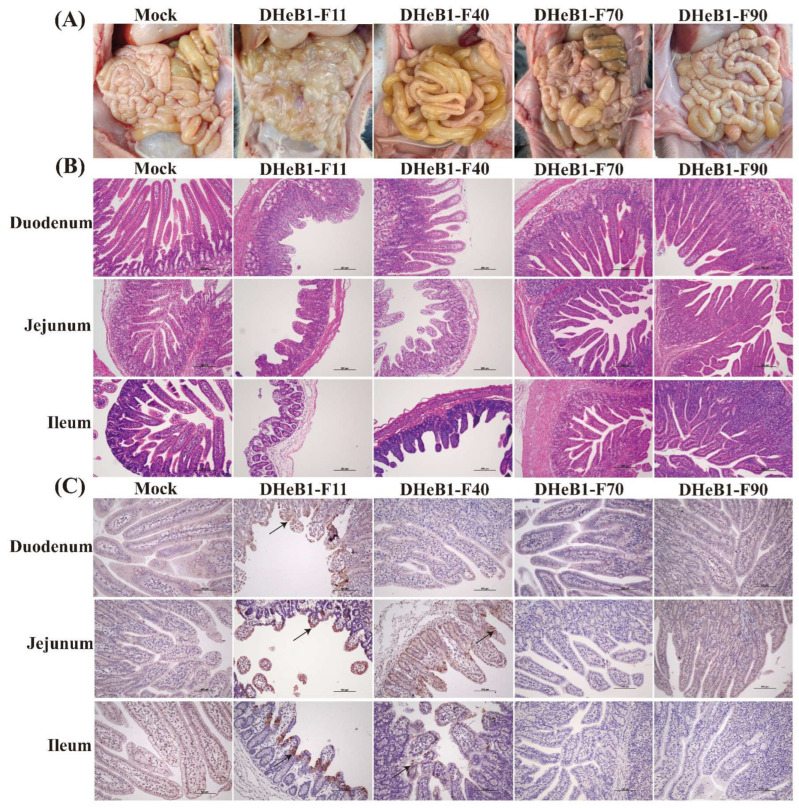
Necropsy, histopathology, and immunohistochemical analysis of piglets inoculated with PDCoV DHeB1 F11, F40, F70, or F90 at 60 hpc. (**A**) Macroscopic lesions of PDCoV DHeB1-challenged and mock-infected piglets at 60 hpc. (**B**) H&E-stained small intestine tissue sections of PDCoV DHeB1-challenged and mock-infected piglets at 60 hpc. (**C**) Immunohistochemically stained small intestine tissue sections of PDCoV DHeB1-challenged and mock-infected piglets at 60 hpc. The arrow indicates the positive signal of the PDCoV N protein.

One piglet from each group was randomly euthanized at 60 hpc for the examination of gross and histopathological lesions. Severe pathological changes were observed in the small intestines of piglets inoculated with PDCoV DHeB1-F11. These piglets exhibited gas-distended small intestines with thin transparent intestinal walls, and the intestinal lumen was filled with large amounts of liquid intestinal contents. In the DHeB1-F40 challenge group, the small intestines of piglets contained yellow fluid and displayed moderately thinned walls. In contrast, piglets inoculated with PDCoV DHeB1-F70 or F90 showed no visible pathological lesions in their small intestines, resembling those of the mock group (Figure 4A). The histopathological analysis results revealed that PDCoV DHeB1 infection caused varying degrees of damage to the small intestines of piglets, characterized by shortened, blunted, and fused intestinal villi, as well as detachment of enterocytes. The most severe lesions were observed in piglets infected with PDCoV DHeB1-F11, which exhibited significant villous atrophy in the duodenum, jejunum, and ileum. Piglets inoculated with PDCoV DHeB1-F40 developed moderate to severe villous atrophy in the jejunum and ileum. In contrast, piglets infected with either PDCoV DHeB1-F70 or F90 displayed histologically normal small intestinal architecture without significant pathological changes, similar to the observations in the mock group (Figure 4B). Immunohistochemical examination indicated that PDCoV-N protein was predominantly located in the cytoplasm of the epithelial cells of the atrophied villi. It was detected in the duodenum, jejunum, and ileum of piglets infected with PDCoV DHeB1-F11, as well as in the jejunum and ileum of piglets inoculated with PDCoV DHeB1-F40. However, the presence of PDCoV-N protein was minimal in the small intestines of piglets inoculated with PDCoV DHeB1-F70 or F90. As anticipated, no PDCoV-N protein was detected in the small intestines of the mock group piglets (Figure 4C). Based on the results of macroscopic, histopathological, and immunohistochemical analyses, our findings indicate that the virulence of PDCoV DHeB1 was significantly attenuated following serial passage in vitro.

### 3.5. Immunogenicity Evaluation of PDCoV DHeB1 Serially Passaged Strains in Weaned Piglets

**Figure 5:**
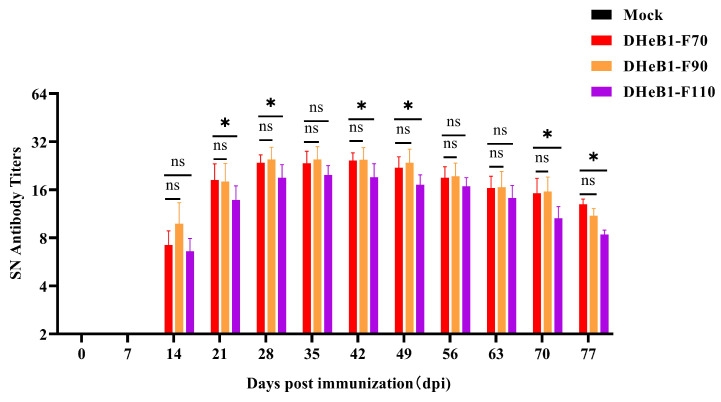
Serum neutralizing antibody titers of weaned piglets immunized with PDCoV DHeB1 serially passaged strains. The weaned piglets were intramuscularly vaccinated with 2 mL (2 × 10^6^ TCID_50_) of PDCoV DHeB1 serially passaged strains (F70, F90, and F110), respectively, and the piglets in the mock group were injected with 2 mL of MEM. Serum samples were collected weekly, and the titers of PDCoV neutralizing antibodies in the serum were detected using a neutralization test (*: *p* < 0.05; ns: *p* > 0.05).

To assess the immunogenicity of PDCoV DHeB1 different passaged strains, weaned piglets were intramuscularly vaccinated with F70, F90, and F110, respectively. Serum samples were collected weekly, and the titer of PDCoV neutralizing antibodies in serum was determined using a neutralization test. As shown in Figure 5, PDCoV neutralizing antibodies in swine sera were initially detected at 14 days post-immunization (dpi) and exhibited a rapid increase, reaching the peak average titer at 28 dpi. Notably, the neutralizing antibody titers induced by PDCoV DHeB1-F70 and F90 were comparable and significantly higher than those induced by F110. No PDCoV neutralizing antibodies were detected in serum samples collected from pigs in the mock group (Figure 5). These results indicate that the immunogenicity of PDCoV DHeB1-F70 and F90 is superior to that of F110, suggesting that excessive passaging in vitro may diminish the immunogenicity of PDCoV DHeB1 in piglets.

## 4. Discussion

The presence of PDCoV was first documented by Woo and his colleagues in Hong Kong in 2012. Subsequently, a PDCoV outbreak in swine herds was reported in the United States in 2014 and has since spread rapidly to numerous countries, including China, Japan, Korea, Thailand, and Vietnam [12,14,29,31,32]. PDCoV primarily infects piglets younger than 7 days old and has emerged as one of the significant pathogens responsible for piglet mortality. Additionally, PDCoV possesses the capability to cross species barriers, infecting a variety of avian and mammalian species [20,33,34]. Previous studies have detected PDCoV in plasma samples from three Haitian children with acute fever, indicating that the virus can infect humans [9]. These findings suggest that PDCoV poses a serious threat to both human and animal health. However, despite this alarming situation, few commercial vaccines for PDCoV have been made available thus far. Currently, several PDCoV vaccine candidates are undergoing development. Yang et al. developed a vector vaccine using attenuated Salmonella Typhimurium, but this vaccine only provided partial immune protection to piglets and failed to fully defend against PDCoV infection [35]. Li et al. demonstrated that mRNA vaccines based on the PDCoV-S protein could induce strong humoral and cellular immune responses in sows, offering effective passive immune protection to piglets [36]. However, the cost of mRNA vaccines remains an ongoing challenge. Live-attenuated vaccines hold promising prospects due to their strong immunogenicity and lower immunization costs. Previous research has shown that the viral pathogenicity to animals can be attenuated through serial passage in vitro [37]. And it is considered a classical method to obtain live-attenuated vaccine candidates via cell passage [38,39]. In this study, PDCoV strain DHeB1 was serially propagated in LLC-PK1 cells for up to 110 passages, and the complete genome sequences of F11, F40, F70, F90, and F110 were sequenced and analyzed. Genome-wide phylogenetic analyses of PDCoV DHeB1 strain and 49 reference PDCoV strains revealed distinct regional characteristics among PDCoV strains. The strain DHeB1 clustered with strains from the Chinese lineage, suggesting a shared evolutionary ancestor. Several amino acid mutations were observed in PDCoV-encoded proteins during in vitro passage, among which the S glycoprotein showed the highest mutation frequency. The PDCoV S glycoprotein is critical for receptor binding, virus entry, and cell membrane fusion [24]. Previous studies have demonstrated that PDCoV undergoes amino acid mutations at positions S162, S396, and S1094 during in vitro passage [6,40], and these mutations were also observed in our passaged strains. The S162 amino acid is a glycosylation site located on the surface of the structural domain of the amino-terminal domain of the S-protein. The absence of glycosylation at this site may potentially diminish the virus’s ability to evade the host’s immune response [41,42]. In addition, we observed the eight and seven amino acid mutations (including S162, S396, and S1094) that emerged in the 40th and 70th passages, respectively, which remained stable throughout subsequent passages. These mutation sites were located in the ORF1a, S, E, and N proteins, which are closely associated with the replication and virulence of coronaviruses [43,44]. This suggests that these mutations may play a crucial role in the viral adaptation to in vitro replication and its pathogenicity. Further investigations, including reverse genetics and animal studies, are necessary to confirm these findings.

Hu et al. demonstrated that the pathogenicity of the PDCoV-OH-FD22 strain in piglets remained unchanged even after 40 passages in LLC-PK1 cells [45], indicating that further investigation into the pathogenicity of subsequent passages of PDCoV in piglets, as well as the identification of related virulence genes, are crucial. In this study, we examined the pathogenicity of PDCoV DHeB1 serially passaged strains in piglets. The results revealed that piglets inoculated with PDCoV DHeB1-F11 exhibited the most severe diarrhea and intestinal damage, followed by those infected with PDCoV DHeB1-F40, which presented moderate macroscopic and microscopic lesions. However, piglets in the DHeB1-F70 and F90 challenge groups did not exhibit significant clinical symptoms throughout the experiment, and no visible intestinal pathological damage was observed. These results indicate that the pathogenicity of PDCoV DHeB1 in piglets was significantly attenuated after 70 passages in vitro.

Based on the above findings, we further compared the immunogenicity of PDCoV DHeB1 serially passaged strains (F70, F90, and F110). The results of the serum neutralization antibody test revealed that PDCoV DHeB1-F70 and F90 induced significantly higher neutralizing antibody titers than F110 in immunized piglets, suggesting their superior immunogenicity compared with F110. This phenomenon may be attributed to the excessive in vitro passage, which diminishes the viral ability to infect host target cells. Consequently, this leads to a reduction in the number of viral particles and viral antigens, ultimately resulting in decreased stimulation of the host immune system. Notably, while the present research offers valuable insights, it is important to acknowledge that the cellular immune response induced by attenuated viruses remains unexplored. Future studies should prioritize this aspect to enhance the comprehensiveness of the research. Additionally, genetic stability is a significant concern for live-attenuated vaccines. Therefore, additional investigations are required to determine whether the attenuated viruses undergo virulence reversion during in vivo passage.

## 5. Conclusions

In conclusion, our animal experiments demonstrated a significant reduction in the virulence of PDCoV DHeB1 following serial passage in vitro. The attenuated PDCoV DHeB1 can be used to develop a live-attenuated vaccine against PDCoV. Moreover, the complete genome alignment of different passaged strains revealed that the eight and seven amino acid mutations, which occurred at the 40th and 70th passages, respectively, remained stable throughout subsequent passages. These mutations may play a critical role in the viral adaptation to in vitro replication and its virulence. These findings of this study will serve as a crucial theoretical foundation for the development of a PDCoV vaccine and for further investigation into its pathogenic mechanisms.

## Figures and Tables

**Table 1:** Mutations of the amino acid in PDCoV DHeB1 serially passaged strains.

Gene	Amino Acid Position	PDCoV-DHeB1 Passage				
		F11	F40	F70	F90	F110
Nsp2	19	CTA (L)	CCA (P) *	CCA (P)	CCA (P)	CCA (P)
Nsp3	831	GGG (G)	GGG (G)	TGG (W)	TGG (W)	TGG (W)
Nsp4	2129	AGG (R)	AGG (R)	ATG (M)	ATG (M)	ATG (M)
Nsp4	2190	CCT (P)	TCT (S)	CCT (P)	CCT (P)	CCT (P)
Nsp4	2486	GGT (G)	GGT (G)	GGT (G)	AGT (S)	GGT (G)
Nsp5	2555	GAC (D)	GAC (D)	GAC (D)	CAC (H)	CAC (H)
Nsp9	3452	GGA (G)	AGA (R)	AGA (R)	GGA (G)	GGA (G)
Nsp12	163	CCA (P)	CCA (P)	CCA (P)	CCA (P)	CAA (Q)
S	29	CCG (P)	CCG (P)	TCG (S)	TCG (S)	TCG (S)
	140	ATC (I)	ATC (I)	ATC (I)	ATC (I)	ATG (M)
	151	AAT (N)	AAT (N)	AAT (N)	AAT (N)	AAG (K)
	160	TTT (F)	TCT (S)	TCT (S)	TCT (S)	TCT (S)
	162	GAC (D)	TAC (Y)	TAC (Y)	TAC (Y)	TAC (Y)
	169	TCT (S)	TCT (S)	CCT (P)	CCT (P)	CCT (P)
	396	AAT (N)	AAG (K)	AAG (K)	AAG (K)	AAG (K)
	488	CTA (L)	CAA (Q)	CAA (Q)	CAA (Q)	CAA (Q)
	552	ACA (T)	ATA (I)	ATA (I)	ACA (T)	ACA (T)
	641	AAA (K)	AAA (K)	AAA (K)	AAA (K)	CAA (Q)
	705	GCT (A)	ACT (T)	ACT (T)	ACT (T)	ACT (T)
	926	AAT (N)	AAT (N)	AAT (N)	TAT (Y)	AAT (N)
	946	TCT (S)	TTT (F)	TTT (F)	TTT (F)	TTT (F)
	947	GCC (A)	GCC (A)	GCC (A)	GCC (A)	ACC (T)
	1067	GCA (A)	GTA (V)	GTA (V)	GTA (V)	GTA (V)
	1094	CTT (L)	CTT (L)	ATT (I)	ATT (I)	ATT (I)
E	19	CTA (L)	CCA (P)	CTA (L)	CTA (L)	CTA (L)
	61	AAG (K)	AAG (K)	GAG (E)	GAG (E)	GAG (E)
N	101	CCG (P)	CCG (P)	TCG (S)	TCG (S)	TCG (S)

* Light pink and deep pink indicate amino acid mutations that emerged in F40 and F70, respectively, which remained stable in subsequent passages.

## Data Availability

All data are available both in the article text and the Supplementary Files.

## Supplementary Materials

The following supporting information can be downloaded at https://www.mdpi.com/article/10.3390/v17050695/s1: Figure S1. Nucleotide homology analysis of PDCoV DHeB1 different-passaged strains with reference strains; Table S1. PDCoV reference strains used in the genetic evolutionary analysis.
