# Supplementary material for: Attenuation of a Virulent Porcine Deltacoronavirus Strain DHeB1 via Serial Passage in LLC-PK1 Cells
Source: Viruses. 2025 May 12;17(5):695. doi: 10.3390/v17050695 (PMC12116136; doi:10.3390/v17050695)
Supplement: Supplementary file 1 [file viruses-17-00695-s001.zip › viruses-3606388-supplementary.pdf]

Figure S1. Nucleotide homology analysis of PDCoV DHeB1 different passaged strains with reference strains.

Percent Identity

Divergence

	1	2	3	4	5	6	7	8	9	10	11	12	13	14	15	16	17	18	19	20	21	22	23	24	25	26	27
1		99.1	99.1	99.0	99.0	99.0	99.0	99.0	99.1	99.1	99.1	99.2	99.2	99.1	99.1	97.4	97.4	98.8	99.1	97.7	97.8	99.1	98.6	99.1	99.0	98.9	99.4
2	0.8		99.8	98.5	98.6	98.6	98.6	98.6	99.8	99.8	99.8	99.8	99.8	99.8	99.8	97.2	97.2	99.4	98.5	97.6	97.6	99.6	99.3	98.7	98.5	98.7	98.9
3	0.7	0.2		98.6	98.6	98.6	98.6	98.7	99.9	99.9	99.8	99.9	99.9	99.9	99.8	97.3	97.2	99.6	98.6	97.6	97.6	99.6	99.3	98.7	98.6	98.7	99.0
4	1.0	1.3	1.3		99.9	99.9	99.9	99.9	98.6	98.6	98.5	98.6	98.6	98.6	98.5	97.3	97.3	98.2	98.7	97.6	97.7	98.6	98.0	99.7	98.9	98.5	98.7
5	1.0	1.3	1.3	0.1		100.0	99.9	99.9	98.6	98.6	98.6	98.6	98.6	98.6	98.6	97.4	97.4	98.3	98.8	97.7	97.7	98.6	98.1	99.7	98.9	98.6	98.7
6	1.0	1.3	1.3	0.1	0.0		99.9	99.9	98.6	98.6	98.6	98.6	98.6	98.6	98.6	97.4	97.4	98.3	98.8	97.7	97.7	98.6	98.1	99.7	98.9	98.6	98.7
7	1.0	1.3	1.2	0.1	0.1	0.1		99.9	98.6	98.6	98.6	98.6	98.6	98.6	98.6	97.4	97.4	98.3	98.8	97.7	97.7	98.6	98.1	99.7	98.9	98.6	98.7
8	1.0	1.3	1.2	0.1	0.1	0.1	0.1		98.6	98.6	98.6	98.7	98.6	98.6	98.6	97.4	97.4	98.3	98.8	97.7	97.7	98.6	98.1	99.8	98.9	98.6	98.8
9	0.7	0.2	0.1	1.3	1.3	1.3	1.3	1.2		99.9	99.9	99.9	99.9	99.8	99.8	97.3	97.3	99.5	98.6	97.6	97.6	99.6	99.3	98.7	98.6	98.7	99.0
10	0.8	0.2	0.1	1.3	1.3	1.3	1.3	1.2	0.1		99.8	99.9	99.8	99.8	99.8	97.2	97.2	99.5	98.6	97.5	97.6	99.6	99.3	98.7	98.5	98.7	99.0
11	0.8	0.2	0.2	1.3	1.3	1.3	1.3	1.3	0.1	0.2		99.9	99.8	99.8	99.8	97.2	97.2	99.5	98.5	97.5	97.6	99.6	99.3	98.7	98.5	98.7	98.9
12	0.7	0.2	0.1	1.3	1.3	1.2	1.2	1.2	0.1	0.1	0.1		99.9	99.8	99.8	97.3	97.3	99.5	98.6	97.6	97.6	99.6	99.3	98.7	98.6	98.8	99.0
13	0.7	0.2	0.1	1.3	1.3	1.3	1.3	1.2	0.1	0.2	0.2	0.1		99.8	99.8	97.3	97.3	99.5	98.6	97.6	97.6	99.6	99.3	98.7	98.6	98.7	99.0
14	0.8	0.2	0.1	1.3	1.3	1.3	1.3	1.2	0.2	0.2	0.2	0.2	0.2		99.8	97.2	97.2	99.5	98.6	97.5	97.6	99.6	99.3	98.7	98.5	98.7	99.0
15	0.8	0.2	0.2	1.3	1.3	1.3	1.3	1.3	0.2	0.2	0.2	0.2	0.2	0.2		97.2	97.2	99.5	98.5	97.5	97.6	99.6	99.3	98.7	98.5	98.7	98.9
16	2.6	2.7	2.6	2.7	2.6	2.6	2.6	2.6	2.6	2.6	2.6	2.6	2.6	2.6	2.6		100.0	96.9	97.4	98.4	98.4	97.3	96.8	97.5	97.3	97.3	97.2
17	2.6	2.7	2.6	2.7	2.6	2.6	2.6	2.6	2.6	2.7	2.7	2.6	2.6	2.6	2.7	0.0		96.9	97.4	98.4	98.4	97.3	96.8	97.4	97.3	97.2	97.2
18	0.8	0.3	0.2	1.4	1.3	1.3	1.3	1.3	0.2	0.2	0.2	0.2	0.2	0.2	0.2	2.7	2.7		98.2	97.2	97.2	99.3	99.0	98.4	98.2	98.4	98.6
19	0.9	1.3	1.3	1.2	1.2	1.2	1.2	1.2	1.3	1.3	1.3	1.3	1.3	1.3	1.3	2.5	2.5	1.4		97.9	97.9	98.6	98.0	98.9	98.9	98.5	99.4
20	2.2	2.3	2.3	2.4	2.3	2.3	2.3	2.3	2.3	2.3	2.3	2.3	2.3	2.3	2.3	1.6	1.6	2.4	2.1		100.0	97.6	97.1	97.7	97.7	97.6	97.5
21	2.2	2.3	2.3	2.3	2.3	2.3	2.3	2.2	2.3	2.3	2.3	2.2	2.3	2.3	2.3	1.6	1.6	2.4	2.0	0.0		97.6	97.1	97.8	97.7	97.6	97.5
22	0.8	0.3	0.3	1.4	1.4	1.4	1.4	1.3	0.3	0.3	0.3	0.3	0.3	0.3	0.3	2.7	2.7	0.4	1.4	2.4	2.3		99.1	98.7	98.6	98.8	98.9
23	1.2	0.6	0.6	1.7	1.7	1.7	1.7	1.7	0.5	0.6	0.6	0.5	0.6	0.6	0.6	3.0	3.0	0.6	1.7	2.7	2.6	0.7		98.2	98.0	98.2	98.4
24	0.9	1.2	1.1	0.3	0.3	0.3	0.3	0.2	1.2	1.2	1.2	1.1	1.2	1.2	1.2	2.5	2.5	1.2	1.1	2.3	2.2	1.3	1.6		99.0	98.7	98.8
25	1.0	1.4	1.3	1.1	1.1	1.1	1.1	1.1	1.3	1.3	1.4	1.3	1.3	1.3	1.3	2.6	2.7	1.4	1.1	2.3	2.3	1.4	1.7	1.0		98.5	98.8
26	1.1	1.2	1.2	1.4	1.4	1.4	1.4	1.4	1.2	1.2	1.2	1.1	1.2	1.2	1.2	2.7	2.7	1.3	1.5	2.4	2.4	1.3	1.6	1.3	1.4		98.7
27	0.6	1.0	1.0	1.2	1.2	1.2	1.2	1.1	1.0	1.0	1.0	0.9	1.0	1.0	1.0	2.7	2.7	1.1	0.5	2.4	2.4	1.1	1.4	1.1	1.1	1.2	
28	0.8	0.2	0.1	1.3	1.3	1.3	1.3	1.2	0.1	0.2	0.2	0.1	0.2	0.2	0.2	2.6	2.6	0.2	1.3	2.3	2.3	0.3	0.4	1.2	1.3	1.2	1.0
29	0.7	0.2	0.1	1.3	1.3	1.3	1.3	1.2	0.1	0.2	0.2	0.1	0.1	0.2	0.1	2.6	2.6	0.2	1.3	2.3	2.3	0.3	0.4	1.2	1.3	1.2	1.0
30	0.7	0.2	0.1	1.3	1.3	1.2	1.2	1.2	0.1	0.2	0.2	0.1	0.1	0.2	0.2	2.6	2.6	0.2	1.3	2.3	2.2	0.3	0.6	1.1	1.3	1.2	1.0
31	1.5	1.6	1.6	1.9	1.9	1.9	1.9	1.8	1.6	1.6	1.6	1.6	1.6	1.6	1.6	2.7	2.7	1.7	2.0	2.6	2.5	1.7	2.0	1.8	1.9	1.8	1.7
32	1.8	1.9	1.8	2.2	2.1	2.1	2.1	2.1	1.8	1.8	1.9	1.8	1.8	1.8	1.8	3.0	3.0	1.9	2.2	2.8	2.8	1.9	2.2	2.0	2.1	2.0	2.0
33	1.8	1.9	1.9	2.2	2.2	2.2	2.2	2.2	1.9	1.9	2.0	1.9	1.9	1.9	1.9	3.0	3.0	2.0	2.3	2.9	2.8	2.0	2.3	2.1	2.2	2.1	2.1
34	0.6	1.0	1.0	1.2	1.2	1.2	1.2	1.1	1.0	1.0	1.0	1.0	1.0	1.0	1.0	2.7	2.7	1.1	0.7	2.4	2.3	1.1	1.4	1.1	1.0	1.2	0.3
35	1.4	1.6	1.6	1.6	1.6	1.6	1.6	1.5	1.6	1.6	1.6	1.6	1.6	1.6	1.6	2.6	2.6	1.7	1.6	2.3	2.3	1.7	2.0	1.5	1.5	1.9	1.6
36	1.4	1.7	1.7	1.7	1.6	1.6	1.6	1.6	1.7	1.7	1.7	1.6	1.6	1.7	1.7	2.7	2.7	1.7	1.6	2.4	2.3	1.7	2.1	1.6	1.5	2.0	1.6
37	0.9	1.3	1.2	1.3	1.3	1.3	1.3	1.2	1.2	1.2	1.3	1.2	1.2	1.2	1.3	2.9	2.9	1.3	1.3	2.5	2.5	1.3	1.7	1.2	1.4	1.4	1.0
38	1.1	0.5	0.5	1.6	1.6	1.6	1.5	1.5	0.5	0.5	0.6	0.5	0.5	0.5	0.5	2.9	2.9	0.6	1.5	2.5	2.5	0.6	0.9	1.5	1.6	1.4	1.2
39	0.9	0.6	0.7	1.4	1.4	1.4	1.4	1.4	0.7	0.7	0.7	0.7	0.7	0.7	0.7	2.8	2.8	0.8	1.2	2.5	2.4	0.8	1.1	1.3	1.5	1.4	1.0
40	0.9	1.2	1.1	0.3	0.3	0.2	0.2	0.2	1.1	1.2	1.2	1.1	1.1	1.2	1.2	2.5	2.5	1.2	1.1	2.2	2.2	1.3	1.6	0.2	1.0	1.3	1.1
41	1.2	1.5	1.4	1.5	1.4	1.4	1.4	1.4	1.4	1.4	1.5	1.4	1.4	1.4	1.5	2.5	2.5	1.5	1.3	2.1	2.1	1.5	1.8	1.4	1.4	1.8	1.3
42	2.8	2.9	2.9	2.9	2.9	2.9	2.8	2.8	2.8	2.9	2.9	2.8	2.8	2.9	2.9	0.6	0.6	2.9	2.8	1.9	1.9	2.9	3.2	2.8	2.9	3.0	3.0
43	2.8	2.9	2.9	2.9	2.9	2.9	2.8	2.8	2.9	2.9	2.9	2.8	2.9	2.9	2.9	0.6	0.6	2.9	2.8	1.9	1.9	2.9	3.2	2.8	2.9	3.0	3.0
44	2.8	2.9	2.9	2.9	2.9	2.9	2.9	2.8	2.9	2.9	2.9	2.8	2.9	2.9	2.9	0.6	0.6	2.9	2.8	1.9	1.9	3.0	3.2	2.8	2.9	3.0	3.0
45	2.6	2.7	2.6	2.7	2.6	2.6	2.6	2.6	2.6	2.7	2.6	2.6	2.6	2.6	2.7	0.0	0.0	2.7	2.5	1.6	1.6	2.7	3.0	2.5	2.6	2.7	2.7
46	2.6	2.7	2.6	2.7	2.6	2.6	2.6	2.6	2.6	2.6	2.6	2.6	2.6	2.6	2.6	0.0	0.1	2.7	2.5	1.6	1.6	2.7	3.0	2.5	2.6	2.8	2.7
47	2.5	2.6	2.5	2.6	2.5	2.5	2.5	2.5	2.5	2.6	2.6	2.5	2.5	2.5	2.6	0.8	0.8	2.6	2.5	1.5	1.5	2.6	2.9	2.5	2.6	2.7	2.6
48	1.2	1.4	1.4	1.4	1.4	1.3	1.3	1.3	1.4	1.4	1.4	1.4	1.4	1.4	1.4	2.6	2.6	1.5	1.2	2.2	2.2	1.5	1.8	1.3	1.3	1.7	1.2
49	1.3	1.5	1.5	1.6	1.6	1.6	1.6	1.5	1.5	1.5	1.5	1.5	1.5	1.5	1.5	2.5	2.6	1.6	1.5	2.2	2.2	1.6	1.9	1.5	1.5	1.9	1.4
50	1.0	1.4	1.4	1.4	1.4	1.4	1.4	1.3	1.4	1.4	1.4	1.3	1.4	1.4	1.4	2.7	2.7	1.5	1.3	2.4	2.4	1.5	1.8	1.3	1.2	1.6	1.1
51	1.4	1.6	1.6	1.6	1.6	1.6	1.6	1.6	1.6	1.6	1.6	1.5	1.6	1.6	1.6	2.6	2.7	1.6	1.6	2.3	2.3	1.6	2.0	1.5	1.6	1.9	1.5
52	1.4	1.6	1.6	1.6	1.6	1.6	1.6	1.6	1.6	1.6	1.6	1.5	1.6	1.6	1.6	2.6	2.7	1.6	1.6	2.3	2.3	1.6	2.0	1.5	1.6	1.9	1.5
53	1.0	1.3	1.2	0.4	0.4	0.4	0.4	0.3	1.2	1.3	1.3	1.2	1.2	1.3	1.3	2.6	2.6	1.3	1.2	2.3	2.3	1.4	1.7	0.3	1.1	1.4	1.2
54	1.5	1.7	1.7	1.7	1.7	1.7	1.7	1.6	1.7	1.7	1.7	1.6	1.6	1.7	1.7	2.8	2.8	1.7	1.7	2.5	2.4	1.8	2.0	1.6	1.7	2.1	1.7
	1	2	3	4	5	6	7	8	9	10	11	12	13	14	15	16	17	18	19	20	21	22	23	24	25	26	27

	28	29	30	31	32	33	34	35	36	37	38	39	40	41	42	43	44	45	46	47	48	49	50	51	52	53	54		
1	99.0	99.0	99.1	98.5	98.2	98.2	99.4	98.5	98.4	99.1	98.7	99.1	99.1	98.7	97.2	97.2	97.2	97.4	97.4	97.5	98.8	98.6	99.0	98.6	98.6	98.9	98.3	1	KP757892.1
2	99.7	99.7	99.7	98.3	98.1	98.0	98.9	98.2	98.1	98.6	99.3	99.3	98.7	98.4	97.0	97.0	97.0	97.2	97.2	97.3	98.4	98.3	98.5	98.2	98.2	98.5	98.1	2	KR150443.1
3	99.7	99.8	99.8	98.3	98.1	98.0	98.9	98.2	98.1	98.7	99.4	99.2	98.7	98.4	97.0	97.0	97.0	97.2	97.3	97.3	98.4	98.3	98.5	98.3	98.3	98.5	98.2	3	KR265851.1
4	98.5	98.5	98.5	98.1	97.8	97.8	98.8	98.3	98.2	98.7	98.2	98.5	99.7	98.5	97.1	97.1	97.1	97.3	97.3	97.4	98.6	98.4	98.6	98.3	98.3	99.5	98.2	4	DHcB1 - F110
5	98.5	98.5	98.5	98.1	97.8	97.8	98.8	98.3	98.3	98.7	98.2	98.6	99.7	98.5	97.1	97.1	97.1	97.4	97.4	97.4	98.6	98.4	98.6	98.4	98.4	99.5	98.2	5	DHcB1 - F90
6	98.5	98.5	98.5	98.1	97.8	97.8	98.8	98.3	98.3	98.7	98.2	98.6	99.7	98.5	97.1	97.1	97.1	97.4	97.4	97.4	98.6	98.4	98.6	98.4	98.4	99.5	98.2	6	DHeB1 - F70
7	98.5	98.5	98.5	98.1	97.9	97.8	98.8	98.3	98.3	98.7	98.2	98.6	99.8	98.6	97.2	97.2	97.1	97.4	97.4	97.5	98.6	98.4	98.6	98.4	98.4	99.5	98.3	7	DHeB1 - F40
8	98.5	98.5	98.6	98.1	97.9	97.8	98.8	98.4	98.3	98.8	98.2	98.6	99.8	98.6	97.2	97.2	97.2	97.4	97.4	97.5	98.7	98.4	98.6	98.4	98.4	99.6	98.3	8	DHeB1 - F11
9	99.8	99.8	99.8	98.3	98.1	98.0	98.9	98.2	98.1	98.7	99.4	99.2	98.7	98.4	97.0	97.0	97.0	97.3	97.3	97.3	98.4	98.3	98.5	98.3	98.3	98.5	98.2	9	KR265853.1
10	99.7	99.7	99.7	98.3	98.1	98.0	98.9	98.2	98.1	98.7	99.4	99.2	98.7	98.4	97.0	97.0	97.0	97.2	97.2	97.3	98.4	98.3	98.5	98.2	98.2	98.5	98.2	10	KR265856.1
11	99.7	99.7	99.7	98.3	98.0	98.0	98.9	98.2	98.1	98.6	99.3	99.1	98.7	98.4	97.0	97.0	97.0	97.2	97.2	97.3	98.4	98.3	98.5	98.2	98.2	98.5	98.1	11	KR265858.1
12	99.8	99.8	99.8	98.3	98.1	98.0	98.9	98.2	98.1	98.7	99.4	99.2	98.7	98.4	97.0	97.0	97.0	97.3	97.3	97.4	98.4	98.4	98.6	98.3	98.3	98.5	98.2	12	KR265859.1
13	99.7	99.8	99.8	98.3	98.1	98.0	98.9	98.2	98.1	98.7	99.4	99.2	98.7	98.4	97.0	97.0	97.0	97.3	97.3	97.3	98.4	98.3	98.5	98.3	98.3	98.5	98.2	13	KR265861.1
14	99.7	99.7	99.7	98.3	98.1	98.0	98.9	98.2	98.1	98.7	99.4	99.2	98.7	98.4	97.0	97.0	97.0	97.2	97.2	97.3	98.4	98.3	98.5	98.3	98.3	98.5	98.2	14	KR265863.1
15	99.7	99.8	99.7	98.3	98.1	98.0	98.9	98.2	98.1	98.6	99.4	99.2	98.7	98.4	97.0	97.0	97.0	97.2	97.2	97.3	98.4	98.3	98.5	98.3	98.3	98.5	98.2	15	KR265865.1
16	97.1	97.1	97.2	97.2	97.0	96.9	97.3	97.3	97.3	97.1	96.9	97.2	97.5	97.4	99.4	99.4	99.4	100.0	100.0	99.1	97.4	97.4	97.2	97.3	97.3	97.3	97.1	16	KU051641.1
17	97.1	97.1	97.2	97.2	97.0	96.9	97.3	97.2	97.3	97.1	96.9	97.2	97.5	97.4	99.4	99.4	99.4	100.0	99.9	99.1	97.4	97.4	97.2	97.3	97.3	97.3	97.1	17	KU051649.1
18	99.4	99.4	99.4	98.0	97.7	97.7	98.5	97.8	97.7	98.3	99.0	98.8	98.4	98.1	96.7	96.7	96.7	96.9	96.9	97.0	98.1	98.0	98.2	97.9	97.9	98.2	97.9	18	KX022605.1
19	98.5	98.5	98.5	98.0	97.8	97.7	99.3	98.3	98.3	98.6	98.2	98.8	98.9	98.6	97.2	97.2	97.2	97.4	97.4	97.5	98.7	98.5	98.7	98.4	98.4	98.7	98.2	19	KY065120.1
20	97.5	97.5	97.5	97.4	97.2	97.1	97.6	97.6	97.6	97.5	97.2	97.5	97.8	97.8	98.1	98.1	98.1	98.4	98.4	98.5	97.7	97.7	97.5	97.6	97.6	97.6	97.4	20	KX834351.1
21	97.5	97.5	97.5	97.4	97.2	97.1	97.6	97.6	97.6	97.5	97.3	97.6	97.8	97.9	98.2	98.2	98.2	98.4	98.4	98.5	97.8	97.8	97.5	97.7	97.7	97.6	97.4	21	KX834352.1
22	99.5	99.5	99.5	98.3	98.1	98.0	98.9	98.2	98.1	98.7	99.2	99.2	98.7	98.5	97.1	97.0	97.0	97.3	97.3	97.4	98.4	98.4	98.5	98.3	98.3	98.5	98.1	22	KY354363.1
23	99.5	99.6	99.4	97.8	97.6	97.5	98.4	97.7	97.6	98.1	99.1	98.7	98.2	97.9	96.5	96.5	96.5	96.8	96.8	96.9	97.9	97.8	98.0	97.8	97.8	98.0	97.7	23	LC260045.1
24	98.6	98.6	98.6	98.2	98.0	97.9	98.9	98.4	98.3	98.8	98.3	98.7	99.8	98.6	97.2	97.2	97.2	97.4	97.5	97.5	98.7	98.5	98.7	98.5	98.5	99.6	98.3	24	MF431743.1
25	98.4	98.4	98.5	98.1	97.9	97.8	99.0	98.4	98.4	98.6	98.2	98.5	99.0	98.6	97.1	97.1	97.1	97.3	97.3	97.4	98.6	98.5	98.7	98.4	98.4	98.8	98.3	25	KY513724.1
26	98.6	98.6	98.6	98.2	98.0	97.9	98.8	98.0	97.9	98.6	98.3	98.6	98.7	98.2	97.0	97.0	97.0	97.2	97.2	97.3	98.3	98.1	98.4	98.1	98.1	98.5	97.8	26	KY513725.1
27	98.9	98.9	98.9	98.2	98.0	97.9	99.6	98.3	98.2	98.9	98.6	98.9	98.8	98.6	97.0	97.0	97.0	97.2	97.2	97.3	98.7	98.5	98.9	98.4	98.4	98.6	98.2	27	MF280390.1
28		100.0	99.9	98.2	98.0	97.9	98.8	98.0	98.0	98.5	99.4	99.1	98.6	98.3	96.9	96.9	96.9	97.1	97.1	97.2	98.3	98.2	98.4	98.1	98.1	98.4	98.1	28	LC260038.1
29	0.0		99.9	98.2	98.0	97.9	98.8	98.1	98.0	98.6	99.5	99.1	98.6	98.3	96.9	96.9	96.9	97.1	97.2	97.2	98.3	98.2	98.4	98.2	98.2	98.4	98.1	29	LC260041.1
30	0.1	0.1		98.2	98.0	97.9	98.8	98.1	98.0	98.6	99.5	99.1	98.6	98.3	97.0	96.9	96.9	97.2	97.2	97.3	98.3	98.3	98.4	98.2	98.2	98.4	98.1	30	LC260042.1
31	1.6	1.6	1.6		99.6	99.5	98.3	97.7	97.6	98.1	97.9	98.1	98.2	97.9	97.0	97.0	97.0	97.2	97.2	97.3	97.9	97.7	97.9	97.7	97.7	98.0	97.5	31	MF642322.1
32	1.8	1.8	1.8	0.4		99.5	98.1	97.4	97.4	97.8	97.7	97.9	98.0	97.7	96.7	96.7	96.7	97.0	97.0	97.1	97.6	97.5	97.7	97.5	97.5	97.8	97.3	32	MF642324.1
33	1.9	1.9	1.9	0.5	0.5		98.0	97.4	97.3	97.8	97.6	97.8	97.9	97.6	96.7	96.7	96.7	96.9	96.9	97.0	97.6	97.4	97.6	97.4	97.4	97.7	97.2	33	MF642325.1
34	1.0	1.0	1.0	1.7	2.0	2.0		98.4	98.3	99.0	98.5	98.9	98.9	98.6	97.1	97.1	97.0	97.3	97.3	97.3	98.7	98.5	99.0	98.4	98.4	98.7	98.3	34	MH715491.1
35	1.6	1.6	1.6	2.3	2.5	2.6	1.5		98.6	98.1	97.8	98.1	98.4	98.9	97.0	97.0	97.0	97.2	97.2	97.3	98.7	98.7	98.3	98.6	98.6	98.2	98.4	35	MK211169.1
36	1.7	1.7	1.6	2.3	2.5	2.6	1.5	1.3		98.0	97.7	98.0	98.4	99.0	97.0	97.0	97.0	97.3	97.3	97.3	98.7	98.7	98.3	98.7	98.7	98.2	98.5	36	MK993519.1
37	1.2	1.2	1.2	1.9	2.2	2.3	1.0	1.8	1.8		98.3	98.6	98.8	98.4	96.9	96.9	96.9	97.1	97.1	97.2	98.4	98.2	98.6	98.2	98.2	98.6	98.0	37	MK625641.1
38	0.5	0.5	0.5	1.9	2.1	2.2	1.3	1.9	1.9	1.5		98.7	98.3	98.0	96.7	96.7	96.7	96.9	96.9	97.0	98.1	98.0	98.1	97.9	97.9	98.1	97.8	38	MT227371.1
39	0.7	0.7	0.7	1.9	2.1	2.2	1.1	1.8	1.8	1.4	1.0		98.7	98.3	97.0	96.9	96.9	97.2	97.2	97.3	98.4	98.3	98.4	98.2	98.2	98.5	98.0	39	MW685623.1
40	1.2	1.2	1.1	1.8	2.0	2.1	1.1	1.5	1.5	1.2	1.5	1.3		98.6	97.3	97.3	97.2	97.5	97.5	97.5	98.7	98.5	98.7	98.5	98.5	99.6	98.3	40	MZ772936.1
41	1.4	1.4	1.4	2.1	2.3	2.4	1.3	1.0	0.9	1.6	1.7	1.6	1.3		97.2	97.2	97.2	97.4	97.4	97.5	99.1	99.1	98.5	99.0	99.0	98.4	98.9	41	OK546242.1
42	2.9	2.9	2.8	3.0	3.2	3.3	2.9	2.9	2.9	3.1	3.1	3.0	2.7	2.8		100.0	100.0	99.4	99.4	98.9	97.2	97.1	97.0	97.1	97.1	97.1	96.8	42	MZ802772.1
43	2.9	2.9	2.8	3.0	3.2	3.3	2.9	2.9	2.9	3.1	3.1	3.0	2.7	2.8	0.0		100.0	99.4	99.4	98.9	97.1	97.1	97.0	97.1	97.1	97.1	96.8	43	MZ802773.1
44	2.9	2.9	2.8	3.0	3.3	3.3	2.9	2.9	2.9	3.1	3.1	3.0	2.7	2.8	0.0	0.0		99.4	99.4	98.9	97.1	97.1	97.0	97.1	97.1	97.0	96.8	44	MZ802774.1
45	2.6	2.6	2.6	2.7	3.0	3.0	2.7	2.6	2.7	2.9	2.9	2.8	2.5	2.5	0.6	0.6	0.6		100.0	99.1	97.4	97.4	97.2	97.3	97.3	97.3	97.1	45	MZ802775.1
46	2.6	2.6	2.6	2.7	3.0	3.0	2.7	2.6	2.7	2.9	2.8	2.8	2.5	2.5	0.6	0.6	0.6	0.0		99.1	97.4	97.4	97.2	97.3	97.3	97.3	97.1	46	MZ802776.1
47	2.5	2.5	2.5	2.6	2.9	3.0	2.6	2.5	2.6	2.8	2.8	2.7	2.4	2.4	1.1	1.1	1.1	0.8	0.9		97.4	97.4	97.3	97.4	97.4	97.4	97.1	47	MZ802777.1
48	1.4	1.4	1.4	2.1	2.3	2.4	1.2	1.2	1.2	1.5	1.6	1.5	1.2	0.9	2.8	2.8	2.8	2.6	2.6	2.5		99.0	98.7	98.9	98.9	98.5	98.7	48	ON968724.1
49	1.5	1.5	1.5	2.2	2.5	2.6	1.4	1.2	1.2	1.7	1.8	1.7	1.5	0.9	2.8	2.8	2.8	2.6	2.5	2.5	1.0		98.5	99.2	99.2	98.3	98.9	49	OP501870.1
50	1.4	1.4	1.4	2.0	2.2	2.3	1.0	1.5	1.6	1.4	1.7	1.5	1.3	1.4	2.9	2.9	2.9	2.7	2.7	2.7	1.2	1.5		98.4	98.4	98.5	98.2	50	OQ551110.1
51	1.6	1.6	1.6	2.3	2.5	2.6	1.5	1.3	1.3	1.8	1.8	1.8	1.5	1.0	2.9	2.9	2.9	2.6	2.6	2.6	1.1	0.8	1.5		100.0	98.3	98.9	51	OR269935.1
52	1.6	1.6	1.6	2.3	2.5	2.6	1.5	1.3	1.3	1.8	1.8	1.8	1.5	1.0	2.9	2.9	2.9	2.6	2.6	2.6	1.1	0.8	1.5	0.0		98.3	98.9	52	OQ790128.1
53	1.3	1.2	1.2	1.9	2.1	2.2	1.2	1.6	1.6	1.3	1.6	1.4	0.3	1.5	2.9	2.9	2.9	2.6	2.6	2.5	1.4	1.6	1.4	1.6	1.6		98.1	53	OQ790129.1
54	1.7	1.7	1.6	2.4	2.6	2.7	1.6	1.4	1.3	1.9	1.9	1.8	1.6	1.1	3.1	3.1	3.1	2.8	2.8	2.8	1.3	1.0	1.7	1.1	1.1	1.7		54	OR672111.1
	28	29	30	31	32	33	34	35	36	37	38	39	40	41	42	43	44	45	46	47	48	49	50	51	52	53	54		

Table S1. PDCoV reference strains used in the genetic evolutionary analysis.

GenBank accession no.	Strain	Year	Country
KP757892.1	CHN-JS-2014	2014	China
KR150443.1	USA/Arkansas61/2015	2015	USA
KR265851.1	USA/Indiana453/2014	2014	USA
KR265853.1	USA/Minnesota/2013	2013	USA
KR265856.1	USA/Illinois272/2014	2014	USA
KR265858.1	USA/NorthCarolina452/2014	2014	USA
KR265859.1	USA/Minnesota159/2014	2014	USA
KR265861.1	USA/Nebraska210/2014	2014	USA
KR265863.1	USA/Ohio445/2014	2014	USA
KR265865.1	USA/Iowa459/2014	2014	USA
KU051641.1	Swine/Thailand/S5011/2015	2015	Thailand
KU051649.1	Swine/Thailand/S5015L/2015	2015	Thailand
KX022605.1	PDCoV/USA/Nebraska145/2015	2015	USA
KY065120.1	CHN/Tianjin/2016	2016	China
KX834351.1	PDCoV/Swine/Vietnam/HaNoi6/2015	2015	Vietnam
KX834352.1	PDCoV/Swine/Vietnam/Binh21/2015	2015	Vietnam
KY354363.1	DH1	2016	Korea
LC260045.1	HKD/JPN/2016	2016	Japan
MF431743.1	SD	2017	China
KY513724.1	CH/Hunan/2014	2014	China
KY513725.1	CH/Jiangsu/2014	2014	China
MF280390.1	CHN-GD-2016	2016	China
LC260038.1	AKT/JPN/2014	2014	Japan
LC260041.1	IWT/JPN/2014	2014	Japan
LC260042.1	MYZ/JPN/2014	2014	Japan
MF642322.1	CHN/GS/2016/1	2016	China
MF642324.1	CHN/GS/2017/1	2017	China
MF642325.1	CHN/QH/2017/1	2017	China
MH715491.1	PDCoV/CHGD/2016	2016	China
MK211169.1	CHN/Sichuan/2017	2017	China
MK993519.1	CHN/Sichuan/2019	2019	China
MK625641.1	CH/JXJGS01/2016	2016	China
MT227371.1	PDCoV/Peru/isolate/2019	2019	Peru
MW685623.1	PDCoV/Haiti/Human/0256-1/2015	2015	Haiti
MZ772936.1	BN	2021	China
OK546242.1	CZ2020	2020	China
MZ802772.1	PDCoV/CBR-1/2016/Thailand	2016	Thailand
MZ802773.1	PDCoV/CBR-2/2016/Thailand	2016	Thailand
MZ802774.1	PDCoV/CBR-3/2016/Thailand	2016	Thailand
MZ802775.1	PDCoV/NKP-1/2016/Thailand	2016	Thailand
MZ802776.1	PDCoV/VUT-1/2016/Vietnam	2016	Vietnam
MZ802777.1	PDCoV/RBR-1/2016/Thailand	2016	Thailand

ON968724.1	CH/LNFX/2022	2022	China
OP501870.1	CHN-HeN06-2022	2022	China
OQ551110.1	CHN-JS-2018	2018	China
OR269935.1	PDCoV/WH/2023	2023	China
OQ790128.1	PDCoV-KQ-Swine-2023	2023	China
OQ790129.1	PDCoV-SN-Swine-2018	2018	China
OR672111.1	CHN-HN-2023	2023	China
